# HEA-Bench: An AI-Agent-Optimized Calculator of High-Entropy Alloy and Oxide Descriptors and Phase-Prediction Rules

**DOI:** 10.3390/ma19143075

**Published:** 2026-07-17

**Authors:** David Fieser, Unmanaa Dewanjee, Anming Hu

**Affiliations:** Department of Mechanical and Aerospace Engineering, University of Tennessee, 1512 Middle Drive, Knoxville, TN 37996, USAahu3@utk.edu (A.H.)

**Keywords:** high-entropy alloys, high-entropy oxides, thermodynamic descriptors, Miedema model, phase-prediction rules, AI agents, Model Context Protocol, reproducible research software

## Abstract

The empirical descriptors of high-entropy alloys and oxides, from the mixing entropy and atomic-size mismatch to the Miedema enthalpies and the Ω, Φ, and φ stability parameters, are quoted in nearly every design study, yet they are reimplemented ad hoc by individual groups, by closed web calculators, and now inside language-model agent frameworks, where fabrication of property values is a documented failure mode. The resulting numbers disagree and cannot be traced or reproduced. We present HEA-Bench, an open calculator in which every descriptor is a closed-form expression over a curated, literature-cited element-property table, with the six canonical phase-prediction rules reported alongside their thresholds and sources rather than as predictions. One calculation core is delivered as a dependency-free Python (version 3.10 or later) library, a zero-install browser application, an offline desktop executable, and a Model Context Protocol server that exposes it to AI agents as deterministic tools, returning every value with its unit, citation key, and version so an agent’s reasoning trace can be audited. The implementation reproduces published per-alloy and per-oxide anchor values to their printed precision and extends to high-entropy oxides in four structure families. The numerical instability of Ω near zero mixing enthalpy is quantified and exposed as a callable check.

## 1. Introduction

High-entropy alloys (HEAs), or multi-principal-element alloys, occupy the near-equiatomic interior of multicomponent phase diagrams, a design space far too large for exhaustive experiment or first-principles calculation [1,2,3]. To pre-screen this space, the field has converged on a compact set of empirical descriptors that act as proxies for the entropic, enthalpic, and geometric driving forces of single-phase solid-solution formation. These are the configurational mixing entropy $\Delta S_{\mathrm{mix}}$ [1], the atomic-size mismatch δ [4], the valence-electron concentration VEC [5], the Miedema mixing enthalpy $\Delta H_{\mathrm{mix}}$ [6,7], the Yang–Zhang stability parameter Ω [8], and the more recent King Φ [9] and Ye φ [10] indices. Threshold rules over these quantities, for example that a single-phase solid solution is likely when Ω≳1.1 and δ≲6.5%, are quoted in essentially every HEA design study [3,4,8,11]. The same logic has been carried into high-entropy oxides (HEOs) [12], where per-sublattice configurational entropy, Shannon-radius size disorder, and the perovskite tolerance factors play the corresponding role in formability screening [13,14,15,16].

Despite this ubiquity, the computation of these descriptors is in poor shape for reproducible research, and three problems recur.

The first is inconsistent reimplementation. Each group reimplements the formulas, and the results disagree, most of all for the Miedema enthalpies, whose parametrization is not unique. The published Takeuchi–Inoue integer table [7], parameters recomputed from elemental properties [6], and dedicated Miedema calculators [17] differ by several kJ mol^−1^ on specific binaries, with the aluminium and manganese pairs among the most sensitive because their hybridization treatment and effective valence vary between compilations. Section 3.5 and Supplementary Table S5 quantify the resulting spread across a panel of alloys. The problem now propagates into autonomous workflows, and recent work makes the demand for a shared implementation concrete. A ReAct agent framework for HEA discovery built its central tool to compute thirteen of these descriptors for a given composition, with the formulas reimplemented by hand in its own codebase and no validation against the primary literature [18]. Its authors observe that language models in isolation cannot verify their proposals because hallucination of property values is common. A knowledge-enhanced design framework published in npj Computational Materials likewise embeds physics-based descriptors beneath its language-model agents [19]. The pattern is established enough that a recent review of language models for high-entropy alloys names model hallucination and the lack of reproducible infrastructure among the field’s central obstacles, and argues that such models deliver value only when embedded in grounded, verifiable workflows [20]. Each of these frameworks needs exactly the layer this work provides, a validated, citable implementation of the descriptors that an agent can call instead of recomputing from recalled formulas. An agent’s arithmetic is reviewed by no one, so an error in a reimplemented descriptor propagates silently into every composition the agent proposes.

The second is that the available calculators are opaque. The web tools that compute these quantities are generally closed pages with no source code, no statement of which parameter table they use, no offline operation, and no validation against the primary literature. A number copied from such a page cannot be traced or reproduced.

The third is a numerical instability. Because $\Omega ={\bar{T}}_{m}\;\Delta S_{\mathrm{mix}}/\left|\Delta H_{\mathrm{mix}}\right|$ diverges as $\Delta H_{\mathrm{mix}}\to 0$, the value of Ω for a near-ideal alloy is highly sensitive to the pair table, and a small shift in one binary can move it by an order of magnitude. A tool that reports a single Ω with no caveat invites over-interpretation.

Two open tools occupy neighboring ground. The Miedema Calculator [17] is a dedicated platform for Miedema formation enthalpies of binary alloys, and HEAPS [21] is a graphical program that computes semi-empirical HEA parameters. Neither covers the oxide descriptors, neither runs as a dependency-free library or fully in a browser, and neither exposes its calculations to autonomous agents. The matminer featurization library [22] includes a Miedema model among hundreds of machine-learning features but is a heavyweight dependency aimed at building feature matrices, not a validated reference calculator with stated thresholds and verdicts. For the high-entropy-oxide formability descriptors we are not aware of any open calculator at all.

HEA-Bench addresses this gap. Every descriptor is a closed-form expression over a curated, literature-cited element-property table, with no trained model and no hidden parameters. The parameter provenance is stated and versioned, the Miedema pair table is taken from a documented source, and the user can override any pair value. The same core is delivered as four surfaces, a dependency-free Python library and command-line tool, a zero-install browser application, a single offline desktop executable, and a Model Context Protocol (MCP) server that exposes the calculator to AI agents as deterministic tool calls, so it suits a scripting workflow, a quick look-up in a browser, an offline laboratory machine, and an agent workflow alike. An automated test keeps the two implementations identical so the surfaces cannot diverge, and for the Ω instability HEA-Bench reports the per-pair values that produce it and lets the user adjust them. The contribution is the calculator itself, a correct and documented offline reference implementation of widely used quantities. It accepts any input element ratios, whether nominal or measured by energy-dispersive spectroscopy in scanning or transmission electron microscopy (SEM-EDS, TEM-EDS), atom probe tomography (APT), or X-ray photoelectron spectroscopy (XPS), which connects the descriptors directly to experimental composition data for high-entropy materials design and discovery.

## 2. Materials and Methods

This section states every quantity the program computes, in the exact form implemented, with its parameter source, and then describes the implementation and the agent interface. A composition is a set of element symbols with amounts, normalized internally to mole fractions $c_{i}$ with $\sum_{i}c_{i}=1$. Compositions may be entered as chemical formulas such as CoCrFeMnNi or Al_0.3_CoCrFeNi, with integer or fractional subscripts, or element by element, and every surface uses the same parser, so the same string produces the same composition everywhere. Elements outside a given data table raise an explicit coverage warning that names the table and the missing elements, never a silent zero or a partial sum, and a descriptor that cannot be computed for a composition is reported as unavailable together with the reason.

### 2.1. Alloy Descriptors

The ideal configurational mixing entropy [1] is

(1) $$\Delta S_{\mathrm{mix}}=-R\sum_{i}c_{i}\ln c_{i},$$

with *R* the gas constant, so an equimolar *n*-element alloy has $\Delta S_{\mathrm{mix}}=R\ln n$. The atomic-size mismatch [4] is

(2) $$\delta =100\sqrt{\sum_{i}c_{i}{\left(1-\frac{r_{i}}{\bar{r}}\right)}^{\;2}},\;\bar{r}=\sum_{i}c_{i}\;r_{i},$$

over the metallic atomic radii $r_{i}$. The mean melting temperature, the valence-electron concentration [5], and the mean Pauling electronegativity are the composition-weighted means ${\bar{T}}_{m}=\sum_{i}c_{i}T_{m,i}$, $\mathrm{VEC}=\sum_{i}c_{i}\;{\mathrm{VEC}}_{i}$, and $\bar{\chi }=\sum_{i}c_{i}\chi_{i}$, and the electronegativity mismatch is $\Delta \chi ={\left(\sum_{i}c_{i}{\left(\chi_{i}-\bar{\chi }\right)}^{2}\right)}^{1/2}$.

The mixing enthalpy uses the regular-melt pair form of Takeuchi and Inoue [7],

(3) $$\Delta H_{\mathrm{mix}}=\sum_{i<j}4\;\Delta H_{ij}\;c_{i}c_{j},$$

where $\Delta H_{ij}$ is the Miedema-model mixing enthalpy of the equiatomic ij binary [6]. The pair table is vendored from matminer [22] and every $\Delta H_{ij}$ can be overridden by the user, which matters because published Miedema compilations disagree on specific pairs (Section 3.5). The Yang–Zhang stability parameter [8] combines Equations (1)–(3) as

(4) $$\Omega =\frac{{\bar{T}}_{m}\;\Delta S_{\mathrm{mix}}}{|\Delta H_{\mathrm{mix}}|}.$$

The Ye φ index [10] measures entropic stabilization against the packing-mismatch penalty,

(5) $$\varphi =\frac{\Delta S_{\mathrm{mix}}-\left|\Delta H_{\mathrm{mix}}\right|/{\bar{T}}_{m}}{|S_{E}|},$$

where $S_{E}$ is the excess entropy of a hard-sphere mixture with the alloy’s atomic diameters, evaluated from the Mansoori–Carnahan–Starling–Leland equation of state [23]. Following Ye’s convention the program evaluates $S_{E}$ at the bcc and fcc packing fractions (0.68 and 0.74) and averages the two, and the packing fraction is exposed as a parameter.

The King Φ index [9] compares the Gibbs energy of the disordered solid solution, $\Delta G_{\mathrm{ss}}=\Delta H_{\mathrm{mix}}-{\bar{T}}_{m}\Delta S_{\mathrm{mix}}$, against the most stable competing binary intermetallic,

(6) $$\Phi =\frac{\Delta G_{\mathrm{ss}}}{-\;|\Delta G_{\max}|}.$$

King evaluates $\Delta G_{\max}$ from Bakker’s binary intermetallic enthalpy model [24] with a stoichiometric allocation across all binaries. HEA-Bench instead takes the most negative raw pair enthalpy in the alloy as $\Delta G_{\max}$, using King’s own assumption that ordered intermetallics have negligible configurational entropy. This documented approximation makes the absolute Φ values systematically larger than King’s published ones for alloys with four or more elements, by roughly a factor of n/2 for an *n*-element alloy, because the raw pair enthalpy is not diluted by King’s stoichiometric allocation. The direction of the bias fixes how the rule may be read. An inflated Φ can only move a composition toward the solid-solution side of the threshold, so a failing Φ is decisive under either convention while a passing Φ near the threshold is not, and the program reports the convention alongside the value rather than presenting the number as King’s.

### 2.2. Empirical Phase-Prediction Rules

Six canonical threshold rules are evaluated, each returning a verdict together with the computed value, the threshold, and the source (Table 1). The rules are weak empirical screens, not phase predictions, and the program presents them as such. The thresholds are the canonical published values, and each is exposed as a parameter so downstream studies can sweep them. Two rows carry caveats. Zhang and co-workers published a joint window in δ and $\Delta H_{\mathrm{mix}}$, and the tabulated rule applies its size branch with the mixing enthalpy always reported alongside, so the joint window can be read off directly. The King verdict inherits the convention of Equation (6) and can err only toward the solid solution.

### 2.3. Miedema Formation Enthalpies

Beyond the pair-table mixing enthalpy of Equation (3), the browser and desktop surfaces evaluate the full Miedema macroscopic-atom model [6] to decompose formation enthalpies. The model assigns each element an adjusted electronegativity $\varphi^{*}$ and a Wigner–Seitz boundary electron density $n_{\mathrm{ws}}$, both derived from elemental data alone, and the chemical interaction of a pair is governed by the interfacial amplitude

(7) $$\Gamma_{AB}=\frac{-P\;{\left(\Delta \varphi^{*}\right)}^{2}+Q\;{\left(\Delta n_{\mathrm{ws}}^{1/3}\right)}^{2}-R_{\mathrm{hyb}}}{\frac{1}{2}\left(n_{\mathrm{ws},A}^{-1/3}+n_{\mathrm{ws},B}^{-1/3}\right)},$$

in which the electronegativity term is attractive, the electron-density mismatch is repulsive, $R_{\mathrm{hyb}}$ is the hybridization correction for transition-metal with non-transition-metal pairs, and *P* and *Q* are the fixed empirical constants of de Boer and co-workers [6]. Because the interaction acts across cell boundaries, compositions enter through surface concentrations $c_{i}^{\;s}=c_{i}V_{i}^{2/3}/\sum_{k}c_{k}V_{k}^{2/3}$ built on the two-thirds-power molar volumes, with the self-consistent volume correction applied. From this building block the program reports the formation enthalpy of the ordered compound, of the disordered solid solution, and of the amorphous phase. The solid-solution value adds to the chemical term an elastic contribution from the continuum size-mismatch treatment of Niessen and Miedema [25] and a structural contribution from lattice-stability data as a function of average valence-electron count [25]. The amorphous value replaces the elastic and structural terms with the topological enthalpy of non-crystalline packing [26]. The decomposition into chemical, elastic, structural, and topological terms is reported per phase, so the user can see which physical contribution dominates a verdict. The model constants and the self-consistent volume correction are given in the Supplementary Methods.

### 2.4. Oxide Descriptors

The oxide module extends the same closed-form approach to high-entropy oxides [12] in four structure families, rock salt *M*O, perovskite *AB*O_3_, fluorite *M*O_2−*x*_, and pyrochlore $A_{2}B_{2}$O_7_. The first step for any oxide composition is oxidation-state assignment, which the program solves by exact charge balance. Each cation is allowed the oxidation states recorded for it in the vendored data table, the total cation charge must equal twice the oxygen content of the formula unit, and the solver searches the state combinations for an exact balance (see the Supplementary Methods). When no balanced assignment exists, the program raises a structured warning naming the cation set and the target charge rather than guessing because a silently wrong oxidation state would corrupt every radius-based descriptor downstream. Shannon effective ionic radii [27] are then selected by the assigned oxidation state, by the coordination number of the crystallographic site in the chosen family, and by spin state where the table distinguishes one, with high spin the documented default.

The configurational entropy of a multi-sublattice crystal is

(8) $$\Delta S_{\mathrm{conf}}=-R\sum_{k}a_{k}\sum_{i}x_{ik}\ln x_{ik},$$

where $a_{k}$ is the number of sites of sublattice *k* per formula unit and $x_{ik}$ the mole fraction of species *i* on it. Sublattices with a single species contribute zero. The program reports Equation (8) both per formula unit and per cation site because the familiar 1.5R high-entropy threshold applies to the per-cation value. The cation size disorder of a sublattice is the ionic analog of Equation (2) with Shannon radii in place of metallic radii [16], and two sublattices combine as $\delta_{r}^{*}={\left(\delta_{A}^{2}+\delta_{B}^{2}\right)}^{1/2}$.

For perovskites $ABX_{3}$ the program evaluates the Goldschmidt tolerance factor [13],

(9) $$t=\frac{r_{A}+r_{O}}{\sqrt{2}\;\left(r_{B}+r_{O}\right)},$$

with $r_{O}=1.40$ Å the six-fold Shannon radius of O^2−^, against the single-phase window 0.92≤t≤1.04 compiled by Manchón-Gordón and co-workers [16], together with the octahedral factor $\mu =r_{B}/r_{O}$ and its stability window 0.414<μ<0.732. It also evaluates the newer Bartel tolerance factor [14],

(10) $$\tau =\frac{r_{O}}{r_{B}}-n_{A}\left(n_{A}-\frac{r_{A}/r_{B}}{\ln(r_{A}/r_{B})}\right),$$

with $n_{A}$ the composition-weighted mean oxidation state of the A site, predicting a perovskite when τ<4.18. Equation (10) is defined only for $r_{A}>r_{B}$, and the program reports that domain limit explicitly instead of returning a sign-flipped value. For fluorite-structured oxides the program implements the predictor of Spiridigliozzi and co-workers [15], the plain sample standard deviation of the constituent cation radii at eight-fold coordination, which predicts an entropy-stabilized single-phase fluorite above 0.095 Å and whose follow-up correlates the same dispersion with the fluorite transition temperature [28]. For pyrochlores $A_{2}B_{2}O_{7}$ it evaluates the radius ratio $r_{A}/r_{B}$ against the stability window $1.46<r_{A}/r_{B}<1.78$ of Subramanian and co-workers [29], below which a defect fluorite is expected. Every oxide verdict is reported with its window and source, with the same weak-screen framing as the alloy rules.

### 2.5. Implementation

**Figure 1:**
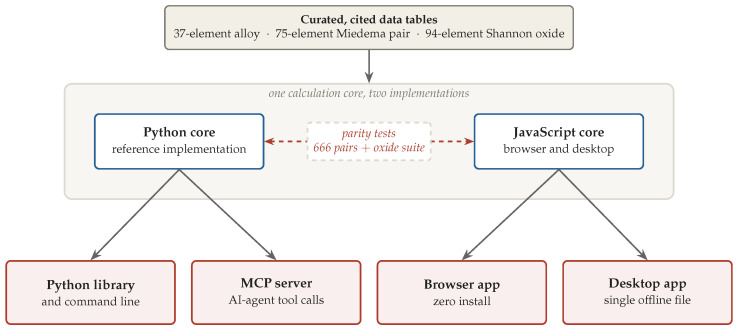
Architecture of HEA-Bench: a reference Python implementation and a parity-tested JavaScript port of one calculation core, delivered as a Python library with a command line, a Model Context Protocol server for AI agents, a zero-install browser application, and an offline desktop application. Solid arrows show the flow of data and computation from the curated tables through the two calculation cores to the four delivery surfaces, and the dashed link marks the automated parity tests that keep the two cores identical. Box color groups the elements by role, the data tables at the top, the two calculation cores in the center, and the four delivery surfaces below.

HEA-Bench is organized around one calculation core with four delivery surfaces (Figure 1). The Python library is the reference implementation. Each descriptor and rule is a function of a composition, given as element symbols with their amounts, which are normalized internally. The library needs no external dependencies, which keeps it installable on air-gapped laboratory machines and inside constrained agent sandboxes alike.

**Figure 2:**
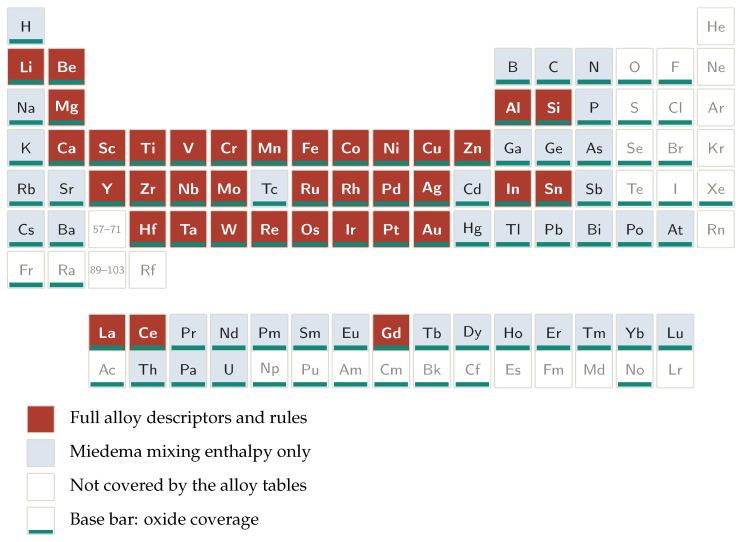
Element coverage. Brick cells have full alloy descriptor and rule support, light blue cells are covered by the Miedema pair-enthalpy table, and a teal base bar marks oxide coverage.

Every quantity is computed over three curated data tables, and the provenance of each is part of the repository. The first is a 37-element table of atomic radius, melting temperature, valence-electron count, and Pauling electronegativity. The metallic radii follow the Goldschmidt twelve-coordinate convention [30,31], the melting points are the CRC Handbook reference values [32], the valence-electron counts follow the transition-metal convention used by Guo and Liu [5], and the electronegativities are the Pauling scale [33]. The complete table of values, element by element, is given in Supplementary Table S6, so a reader can reproduce any descriptor by hand and compare it against their own source data. The second is a 75-element table of Miedema binary mixing enthalpies taken from matminer [22] under the BSD-3-Clause license, chosen deliberately so that values agree with the most widely used machine-learning featurization ecosystem. The third is a 94-element table of Shannon effective ionic radii, oxidation states, and electronegativities for the oxide module, extracted from the pymatgen library’s [34] digitization of the Shannon tables [27] under the MIT license. Each vendored table ships with a provenance file recording the source, the extraction script, the license, and a content hash, and the tables are regenerated only by the recorded scripts, never edited by hand. The JavaScript copies of the tables are themselves generated from the Python tables by sync scripts, so there is exactly one editable copy of every parameter in the project. Figure 2 shows the element coverage across the three tables, and the full element lists by tier are given in Table S1.

Two implementations of one calculation core invite divergence, the standard failure mode of dual-language scientific software, so the two cores are locked together by an automated parity suite that runs on every change. For the alloy core, the suite compares the Python and JavaScript values of every descriptor on all 666 binary pairs of the 37 supported elements and on a set of multi-element fixtures, asserting agreement to a relative tolerance of $5\times 10^{-4}$ and an absolute tolerance of $5\times 10^{-6}$, which absorbs the fixed-precision rounding of the generated tables. For the oxide module, a curated suite of reports covering all four structure families is compared field by field, down to byte-identical warning messages, so even the error paths cannot diverge. The literature anchor values of Section 3.1 are pinned in the same regression suite, so a change that moves a published number fails the build. Parity establishes only that the two implementations agree with each other, not that they are right, so correctness is established separately, against the published anchors of Section 3.1. The version 2 release runs 192 automated tests, releases are versioned semantically with one version number shared by every surface, and the software is archived under a Zenodo concept DOI that always resolves to the latest release. Within this development workflow, the code, the scripts that regenerate the data tables and produce the figures, and the validation and benchmark analyses reported here were written and revised with the assistance of AI coding tools, namely Anthropic’s Claude accessed through the Claude Code command-line tool. Every value they produced was verified by the authors against the tests and anchors above and is reproducible from the shipped software and the cited sources, and the full disclosure of AI use is given in the Acknowledgments.

**Figure 3:**
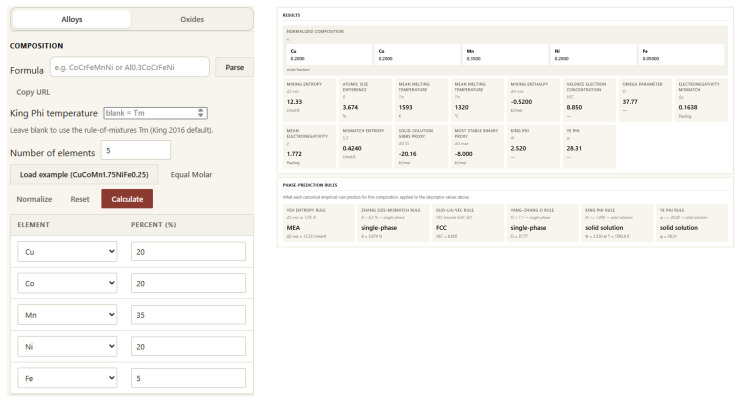
The browser calculator on Co_20_Cu_20_Fe_5_Mn_35_Ni_20_. Left, the composition input with the alloy and oxide modes. Right, the descriptor cards and the six rule verdicts.

The browser application is a self-contained web page that uses the same calculation core and data tables as the library and additionally computes the Miedema formation enthalpies of Section 2.3 with their chemical, elastic, structural, and topological terms. It runs with no server and no network access. The desktop application packages the browser version into a single offline file of about 13 MB using the operating system’s built-in web view. A composition is entered as a chemical formula or element by element, and every numeric card carries a plain-language tooltip stating what the quantity is and which threshold applies (Figure 3). Compositions can be shared as URLs, results can be exported as CSV, any Miedema pair enthalpy can be overridden individually, custom elements can be defined, and the application ships an in-app Theory view that derives every formula with citations to the primary literature.

### 2.6. The Agent Interface

Language-model agents are an emerging consumer of these calculations. The agent literature identifies fabricated property values as a central failure mode [18], and the review literature asks for workflows whose outputs are grounded in verifiable systems rather than recalled from model weights [20]. HEA-Bench fills that role through an MCP server, installed as an optional extra, that exposes the Python core to any MCP-capable agent as seven deterministic tools: composition parsing, batch descriptor evaluation, batch rule evaluation, an Ω sensitivity analysis (Section 3.5) as a callable check, the oxide family reports, an element-coverage query, and a version and citation card. The batch tools accept lists of compositions, because agents sweep candidates and one call evaluating fifty compositions is cheaper and more reliable than fifty calls. Errors are returned as structured messages with recovery hints rather than as exceptions.

**Figure 4:**
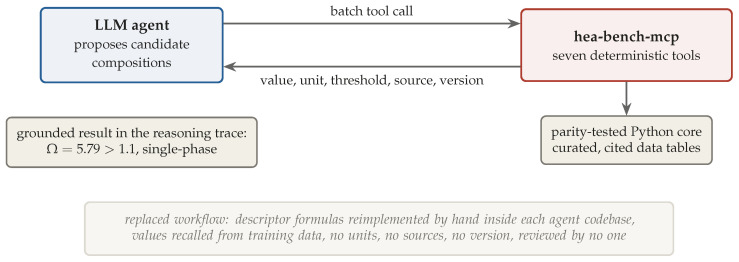
Grounding loop of the agent surface. Batch tool calls return each value with its unit, threshold, citation key, and software version.

The design choice that distinguishes the server is provenance in-band. Every returned value carries its unit, the citation key of its parametrization, and the software version, so the numbers an agent quotes in its reasoning trace can be audited rather than taken on trust (representative responses are given in the Supplementary Materials). Figure 4 shows the resulting grounding loop and what it replaces. A reviewer of an agent-generated design, or the agent itself, can check the margin between a value and its threshold, look up the citation key, and reproduce the number on any of the other surfaces at the stated version. The tool bodies are plain functions with no protocol dependency, unit-tested in the same continuous-integration suite as the library, and the protocol layer is a thin wrapper, so the agent surface inherits the parity guarantees of the core.

## 3. Results

### 3.1. Validation Against Literature Anchors

The implementation is validated against published per-alloy and per-oxide values, and every anchor is pinned in the regression suite so it cannot drift. The primary alloy anchor is Table 1 of Yang and Zhang [8], the paper that defines Ω. Table 2 compares the published values with the calculator’s output for the eight compositions of that table with complete rows. The mixing enthalpies agree to the two decimals Yang and Zhang print, which confirms both that the vendored pair table reproduces the Takeuchi–Inoue parametrization they used [7] and that Equation (3) is implemented identically. The size mismatches differ by at most 0.31 percentage points and the Ω values by at most 2.2 %, and both residuals have documented causes rather than free parameters: δ inherits the atomic-radius compilation and Ω inherits the elemental melting points through ${\bar{T}}_{m}$. The equimolar Cantor alloy CoCrFeMnNi completes the alloy anchors as a smoke test. The calculator returns a mixing entropy of exactly Rln5, a valence-electron concentration of 8, and all six rule verdicts consistent with the single-phase fcc solid solution observed experimentally [2].

The oxide module is anchored against several independent published results, spanning three structure families and both sides of a formability threshold. For the rock-salt entropy-stabilized oxide of Rost and co-workers [12], (Mg,Co,Ni,Cu,Zn)O, the charge-balance solver assigns all five cations their divalent states and Equation (8) returns exactly Rln5 per cation. For the three fluorite-structured entropy-stabilized oxides characterized by Spiridigliozzi and co-workers, the calculator solves the oxidation states by charge balance, selects the eight-fold Shannon radii, and reproduces the published cation-radius standard deviations of all three compositions to every quoted digit [28]. These three rows are the discriminating oxide anchors, because the quoted digits depend on the whole pipeline behind them: a wrong oxidation state, coordination number, or spin-state default would move the third decimal. The fluorite screen is also anchored on the other side of its window: the five-cation oxide (Ce,Zr,Hf,Sn,Ti)O_2_ of Chen and co-workers [35], which is single-phase only at high temperature and reverts to multiple phases when annealed lower, computes σ=0.0835, below the 0.095 Å threshold, consistent with its observed reluctance to remain single-phase. For the single-phase high-entropy perovskite of Jiang and co-workers [36], Sr(Zr,Sn,Ti,Hf,Mn)O_3_, it assigns all five B-site cations their tetravalent states and places the composition inside the reported single-phase window on both the Goldschmidt and Bartel factors [14]. Beyond this single high-entropy composition, the perovskite tolerance-factor implementation was checked against the 576 experimentally characterized $ABX_{3}$ perovskites and non-perovskites tabulated by Bartel and co-workers [14]: it reproduces their published Goldschmidt *t* to a mean absolute deviation of $3\times 10^{-3}$, and the τ<4.18 criterion recovers the experimental label with an accuracy of 0.917, matching the figure reported in that work and agreeing with its own published predictions on every row. The same reports, including the warning messages, are produced identically by the Python and JavaScript implementations. Table 3 collects the anchors.

### 3.2. Empirical Screens and Descriptors on Public Labeled Data

The anchors above establish that the calculator reproduces the published quantities. A separate question, prompted by the empirical rules themselves, is how far those rules agree with observed phases when they are applied as classifiers. HEA-Bench presents them as weak screens throughout, and that framing can be made quantitative. We evaluated the shipped rules against a public labeled union of 7127 compositions assembled from three open datasets of experimentally observed high-entropy-alloy phases [37,38,39], scoring each rule that separates single-phase solid solutions from multi-phase microstructures against the observed label (Supplementary Table S3). The canonical thresholds recover most single-phase alloys but also admit many multi-phase ones, so a single rule separates the two classes only weakly, in line with the known degradation of these criteria away from the datasets on which they were fitted [11]. That is the quantitative basis for the design choice running through the tool, which reports every rule with its threshold and source rather than as a prediction.

The descriptors that feed those rules carry more signal than any single threshold extracts from them, which is why recent machine-learning phase classifiers are built on exactly the quantities the calculator computes. Mandal and co-workers train six standard classifiers on the atomic-size mismatch, the electronegativity mismatch, the valence-electron concentration, and the Miedema mixing enthalpy and entropy, reaching about 94% phase accuracy and finding the atomic-size mismatch the most influential feature [40]. Using HEA-Bench to compute those same five descriptors on a public labeled dataset [38] and feeding them to off-the-shelf classifiers reproduces both effects, a trained model that scores well above any single-rule screen and an atomic-size mismatch ranked first in importance (Supplementary Table S4). HEA-Bench is thus the verified, version-stamped descriptor layer that such models consume, consistent with its positioning beneath trained classifiers rather than as a competing predictor.

### 3.3. Descriptor-Space Map

**Figure 5:**
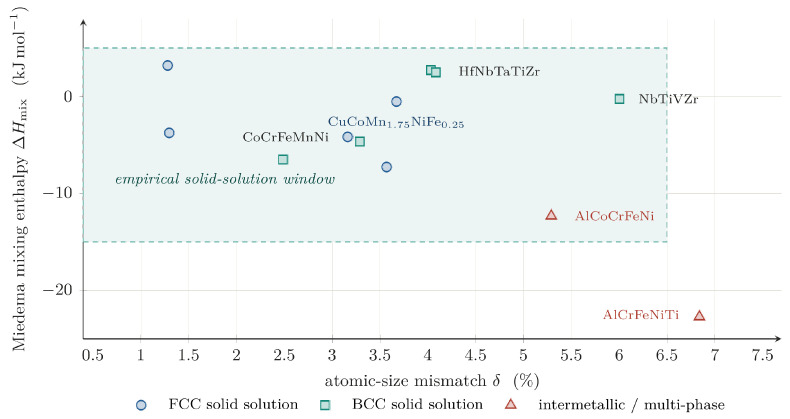
Atomic-size mismatch and Miedema mixing enthalpy for twelve representative high-entropy alloys, computed with the Python library. The dashed region is the empirical solid-solution window of Zhang and co-workers [4].

Figure 5 places twelve well-known high-entropy alloys in the descriptor space the tool produces, with every value computed by the Python library. The picture is the familiar one. The fcc and bcc solid solutions sit inside the empirical window of Zhang and co-workers [4], with the refractory bcc alloys at larger size mismatch, while the aluminum-bearing intermetallic-forming compositions fall outside it at strongly negative mixing enthalpy. Reproducing this canonical map from the shipped tables is itself a useful sanity check, because it confirms that the vendored parametrization recovers the qualitative structure of the field’s standard screening plot, not merely individual anchor values.

### 3.4. Oxide Formability Screens

**Figure 6:**
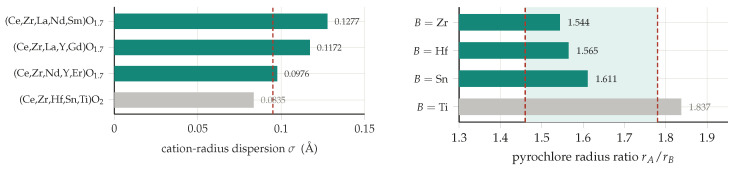
Oxide formability screens computed by the library. (**Left**) the fluorite radius-dispersion screen [15] with the 0.095 Å threshold dashed. The three single-phase compositions are the entropy-stabilized oxides of Spiridigliozzi and co-workers [28], and the below-threshold case (Ce,Zr,Hf,Sn,Ti)O_2_ is the oxide of Chen and co-workers [35], single-phase only at high temperature. (**Right**) the pyrochlore radius-ratio window [29] for (La,Ce,Nd,Sm,Eu)_2_*B*_2_O_7_ with varied B site. In both panels a teal bar marks a composition that falls inside the formability window and a gray bar one that falls outside it, and the dashed red lines mark the bounds of the window.

Figure 6 shows the two radius-based screens in action, with every number computed by the library from the cation lists alone. In the fluorite panel, the three rare-earth compositions of Spiridigliozzi and co-workers sit above the 0.095 Å dispersion threshold and match the published standard deviations digit for digit, while a transition-metal mixture with much more uniform radii falls below the window. In the pyrochlore panel, walking the B site of a five rare-earth $A_{2}B_{2}$O_7_ through Zr, Hf, Sn, and Ti shows the radius-ratio window discriminating: the first three land inside it and Ti falls above, where no single cubic phase is expected [29].

### 3.5. The Ω Instability as a Worked Example

**Figure 7:**
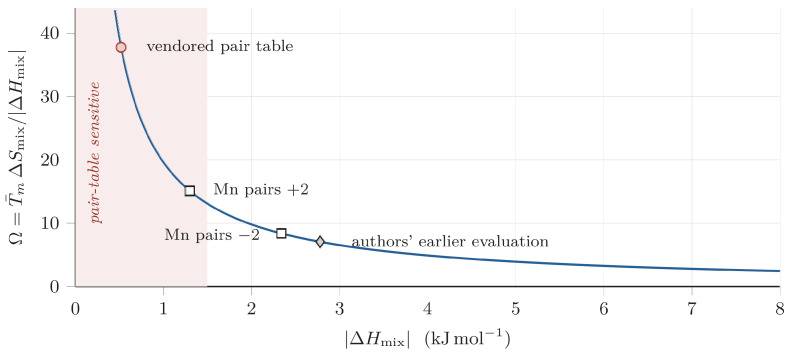
Ω as a function of $|\Delta H_{\mathrm{mix}}|$ for Co_20_Cu_20_Fe_5_Mn_35_Ni_20_ at fixed ${\bar{T}}_{m}$ and $\Delta S_{\mathrm{mix}}$. The circle is the vendored pair table, the squares shift each manganese pair enthalpy by ±2 kJ mol^−1^, and the diamond is the authors’ earlier Miedema evaluation of the same alloy [42,43]. The shaded band is the near-singular region $|\Delta H_{\mathrm{mix}}|\;<1.5$ kJ mol^−1^, where Ω is most sensitive to the pair table.

**Figure 8:**
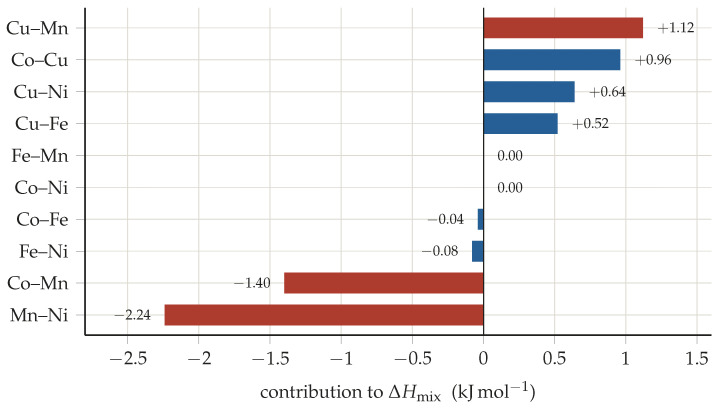
Pair contributions $4\;\Delta H_{ij}\;c_{i}c_{j}$ to the mixing enthalpy of Co_20_Cu_20_Fe_5_Mn_35_Ni_20_. Manganese-containing pairs are shown in red and the remaining pairs in blue.

The near-ideal alloy Co_20_Cu_20_Fe_5_Mn_35_Ni_20_ comes from our earlier experimental work on laser-synthesized high-entropy-alloy nanoparticles, where it is written CuCoMn_1.75_NiFe_0.25_ [41,42,43]. It exposes the Ω instability and is the composition shown in Figure 3. Its entropy and melting temperature are essentially independent of the parameter table, but its mixing enthalpy is nearly zero, so the vendored pair table puts Ω near 38 while sitting almost at the singularity of Equation (4). The per-pair readout (Figure 7 and Figure 8) shows why this number must be read with care. The manganese pairs supply almost all of the enthalpy (Table S2), and published Miedema compilations disagree by several kJ mol^−1^ on precisely these pairs [6,7,17]. Shifting each manganese pair enthalpy by ±2 kJ mol^−1^, a perturbation within that spread, collapses Ω to between 8 and 15, and our own earlier Miedema evaluation of this alloy, with a different parametrization, reported Ω=7.07 [42,43] and falls on the same curve. The order-of-magnitude swing is a property of the metric near $\Delta H_{\mathrm{mix}}=0$, not of the alloy, and every variant returns the same single-phase verdict. HEA-Bench warns whenever the mixing enthalpy is near zero and lets the user enter the pair values of any specific compilation to reproduce it exactly. This analysis is also packaged as one of the agent tools (Section 2.6): given a composition, the tool returns the pair contributions of Figure 8, identifies the dominant element, and reports the Ω interval under a user-chosen pair perturbation, flagging the case where the interval crosses zero mixing enthalpy and the metric diverges.

### 3.6. A Grounded Agent Workflow

The grounding loop of Figure 4 is best shown on the case the previous section quantified. In the call sequence a screening agent makes, one batch call evaluates the Yang–Zhang rule for the Cantor alloy and the near-ideal alloy together. Both pass, but with sharply different reported margins: the Cantor alloy returns Ω=5.79 and the near-ideal alloy returns Ω=37.77, so ranked on the reported margin the second composition looks more than six times as stable, and an agent that recalls or reimplements Ω on its own has no reason to doubt that. A second call, to the sensitivity tool, tests the margin. For the near-ideal alloy it reports the mixing enthalpy as −0.52 kJ mol^−1^, identifies manganese as the dominant element, and returns the Ω interval [8.39, 15.11] under a ±2 kJ mol^−1^ manganese perturbation, with a divergence flag because the perturbation interval crosses zero mixing enthalpy. The same call on the Cantor alloy returns a bounded interval, Ω between 4.43 and 8.37 with no divergence flag, so the smaller of the two margins is in fact the trustworthy one. The exchange costs the agent two round trips, and every number in it can be reproduced on any surface at the stated version. It separates a reliable margin from an artifact of the pair table, a distinction that the bare Ω magnitude does not expose. The corresponding raw tool responses are reproduced in the Supplementary Materials. We ran the corresponding with-and-without comparison directly. Given the same near-ideal alloy and asked to compute the full descriptor set, a language model (Claude Haiku 4.5) working unaided fabricated the values that require the pair table or a competing-phase model, its mixing enthalpy and Ω each being wrong by more than an order of magnitude, and it reported no sign of the numerical instability, whereas the same model connected to the tool returned every value exactly and surfaced the divergence (Supplementary Table S7). Extending this into a systematic bare-against-grounded benchmark across many compositions, measuring the rate of fabricated values and verdict flips, is the natural next step and is left for future work.

## 4. Discussion

HEA-Bench shares basic functionality with two earlier programs. The Miedema Calculator [17] is the reference platform for Miedema formation enthalpies of binary alloys, with a physics depth on that one model that HEA-Bench does not attempt to replicate. HEAPS [21] computes a broad set of semi-empirical HEA parameters through a graphical interface. HEA-Bench differs from both in scope and in engineering (Table 4). It is the only one of the three that covers high-entropy oxides, that runs with zero installation in a browser or as a dependency-free Python library, that pins its outputs and literature anchors in an automated test suite with a dual-implementation parity lock, and that exposes its calculations to AI agents. Conversely, HEAPS offers interactive composition-range exploration that HEA-Bench does not, and the Miedema Calculator implements extensions of the Miedema model beyond our scope, so the three tools are complementary.

Against general-purpose featurization, matminer [22] computes Miedema enthalpies and many related quantities as machine-learning features, and HEA-Bench vendors its Miedema pair data precisely to stay consistent with that ecosystem. The difference is intent. A featurizer produces matrices for model training inside a large dependency stack, whereas HEA-Bench produces individually traceable values with thresholds, verdicts, warnings, and citations, in environments as thin as a single offline executable. The distinction has a reproducibility consequence that goes beyond convenience. When the same descriptor names are computed from different pair tables in different studies, models trained on one dataset are silently inconsistent with features computed for another, a failure mode that no amount of downstream rigor can repair. Pinning the parametrization, stamping the version into every result, and exposing the per-pair values removes that ambiguity at the source.

In practice, the tool is a fast, transparent pre-screen and a sanity check on more expensive CALPHAD or DFT calculations, and a dependable feature generator for downstream data-driven models, since the descriptors are exactly the features most HEA machine-learning studies use [18,22]. Because all six rule verdicts are reported side by side with their values and thresholds, disagreement among the rules is visible rather than hidden behind one aggregated answer, and that disagreement is itself informative, since compositions sitting near several thresholds at once are precisely the ones where the empirical screens are least reliable [11]. We stress what the program does not do. The empirical rules it implements are weak screens whose published thresholds were fitted to particular alloy datasets and are known to degrade outside them [11], and HEA-Bench reports them with sources rather than claiming predictive performance. Trained machine-learning classifiers outperform these rules at phase classification, as quantified in Section 3.2 and reported by recent studies built on these same descriptors [40], and agent frameworks increasingly train their own surrogates [18,19]. HEA-Bench is positioned beneath such models as the verified, auditable descriptor layer, not as a competing predictor.

The remaining limitations are stated plainly. The program ships one Miedema parametrization, chosen for consistency with matminer, and treats the disagreement between compilations as a quantified sensitivity rather than resolving it, since no resolution exists in the literature. The King Φ implementation uses the documented pair-enthalpy approximation of Section 2.1 rather than Bakker’s full binary model, so its absolute scale differs from the original paper. The oxide module covers four structure families and treats the oxygen sublattice as ordered. The families it leaves out are excluded for a principled reason rooted in the same determinism as the rest of the tool, which assigns oxidation states by exact charge balance and then selects a Shannon radius for each cation without guessing. The spinel family is the clearest case. Its cations partition over tetrahedral and octahedral sites, and the degree of inversion is a continuous order parameter that charge balance does not fix, because normal, inverse, and intermediate spinels all satisfy the same cation charge. That parameter is set by octahedral site-preference energies, temperature, and synthesis route, so both the configurational entropy and the size-disorder descriptor of a spinel depend on a site distribution the calculator cannot determine from composition alone. Reporting one would require the very kind of guess the pipeline is built to avoid. Single-phase high-entropy spinels are well documented [44], and a spinel module would need an explicit inversion model rather than a radius lookup. Strongly non-stoichiometric and mixed-valence oxides fall outside for the same reason. A variable oxygen deficit and a fractional average valence, such as coexisting Ce^3+^ and Ce^4+^ or Mn^3+^ and Mn^4+^ on a single element, have no single integer charge balance and no single Shannon radius, and vacancy disorder adds anion-sublattice entropy that the ordered-oxygen assumption omits. The boundary is precise rather than a blanket exclusion of non-stoichiometry. Fixed, writable, charge-balanced non-stoichiometry is already handled, and the fluorite oxide (Ce,Zr,Nd,Y,Er)O_1.7_ is an anchor. Because the calculation core is decoupled from the interfaces and parity-tested, the project is a stable foundation for closing these gaps, and for a planned comparison layer that runs published phase-prediction models side by side on a common benchmark.

## 5. Conclusions

HEA-Bench packages the standard high-entropy alloy and oxide descriptor calculations and phase-prediction rules as open, validated, reproducible research software, delivered as one calculation core behind four interchangeable surfaces, with the two core implementations guaranteed identical by an automated parity suite. It replaces ad hoc reimplementations and opaque web calculators with a transparent, citable reference, it reproduces published per-alloy and per-oxide anchor values to their printed precision, and it reports rather than hides the parametrization sensitivity of the Miedema-based metrics, turning the Ω instability from a recurring source of confusion into a quantified, callable check. To our knowledge it is the first open calculator of the high-entropy-oxide formability descriptors, and we are not aware of another materials tool whose agent interface returns units, citation keys, and the software version with every value. As language-model agents move from demonstrations into routine alloy and oxide design, a validated descriptor layer that any agent can call is enabling infrastructure the field will need regardless of which agent framework prevails, and a systematic measurement of how much such grounding improves agent proposals is left to future work. Grounding those workflows in citable, version-stamped arithmetic is the contribution this program is built around. The software is MIT-licensed, dependency-free at its core, fully offline, and archived for citation.

## Figures and Tables

**Table 1:** The six empirical rules as implemented. Each verdict is reported with the computed value, the threshold, and the citation.

Rule	Criterion	Verdict
Yeh entropy [1]	$\Delta S_{\mathrm{mix}}>1.5R$	high-entropy class
Zhang size [4]	δ<6.5% (size branch)	single-phase solid solution
Guo–Liu VEC [5]	VEC≥8.0/VEC<6.87	fcc/bcc
Yang–Zhang [8]	Ω>1.1	single-phase solid solution
King [9]	Φ>1.0	solid solution over intermetallic
Ye [10]	φ>20	solid solution over intermetallic

**Table 2:** Alloy anchors: the complete rows of Table 1 of Yang and Zhang [8] against the values computed by HEA-Bench. $\Delta H_{\mathrm{mix}}$ in kJ mol^−1^, δ in %. The δ and Ω residuals trace to the atomic-radius and elemental melting-point compilations. Every row is pinned in the regression suite.

	Δ*H*_mix_		*δ*		Ω	
Alloy	Publ.	This Work	Publ.	This Work	Publ.	This Work
CoCrFeNiMo_0.3_	−4.15	−4.15	2.92	2.84	5.97	6.00
CoCrFeNiAl_0.3_Mo_0.1_	−7.26	−7.26	3.74	3.77	3.37	3.39
CoCrFeNiCuAlMo_0.2_	−4.47	−4.47	4.95	4.95	5.70	5.82
Ti_0.8_CoCrFeNiCu	−6.75	−6.75	5.26	5.56	3.95	3.95
TiCoCrFeNiCu	−8.44	−8.44	5.65	5.96	3.17	3.17
Ti_1.5_CoCrFeNiAl	−23.91	−23.91	6.93	7.10	1.08	1.08
CoCrFeNiCuAlMn	−5.63	−5.63	4.57	4.79	4.61	4.64
CrFeNiCuZr	−14.40	−14.40	9.91	9.94	1.70	1.71

**Table 3:** Oxide and smoke-test anchors with the corresponding computed values. The per-alloy descriptor anchors are in Table 2. The fluorite screen is anchored both above its window (the three Spiridigliozzi oxides) and below it (the Chen oxide), and the perovskite tolerance factor is additionally validated over 576 published $ABX_{3}$ compounds. Every single-composition row is pinned in the regression suite.

Anchor	Quantity	Published	Computed
CoCrFeMnNi [2]	phase	single-phase fcc	single-phase fcc, all six rules
(Mg,Co,Ni,Cu,Zn)O [12]	oxidation states	all 2+	all 2+
(Mg,Co,Ni,Cu,Zn)O	$\Delta S_{\mathrm{conf}}$ per cation	Rln5	Rln5 (exact)
Sr(Zr,Sn,Ti,Hf,Mn)O_3_ [36]	Goldschmidt *t*	0.92≤t≤1.04	0.979
Sr(Zr,Sn,Ti,Hf,Mn)O_3_	Bartel τ [14]	τ<4.18	3.72
(Ce,Zr,Nd,Y,Er)O_1.7_ [28]	σ (Å)	0.0976	0.0976
(Ce,Zr,La,Y,Gd)O_1.7_	σ (Å)	0.117	0.1172
(Ce,Zr,La,Nd,Sm)O_1.7_	σ (Å)	0.128	0.1277
(Ce,Zr,Hf,Sn,Ti)O_2_ [35]	σ (Å), below window	σ<0.095 (multiphase)	0.0835
576 $ABX_{3}$ [14]	τ<4.18 accuracy	≈0.91	0.917

**Table 4:** Capability comparison with the closest existing tools, as described in the cited publications. An entry of n/a marks a capability not described in the cited publication.

Capability	HEA-Bench	HEAPS [21]	Miedema Calc. [17]	Matminer [22]
Alloy descriptors and rule verdicts	yes	yes	enthalpies only	features, no verdicts
Miedema formation enthalpies	yes	n/a	yes	yes
High-entropy-oxide descriptors	yes	no	no	no
Zero-install browser use	yes	no	no	no
Dependency-free library	yes	no	no	large dependency stack
Parity-locked dual implementation	yes	n/a	n/a	n/a
AI-agent interface	yes	no	no	no

## Data Availability

The software, the curated and vendored element-property data tables with their provenance documentation and licenses, and the full test suite are openly available in the repository at https://github.com/dfieser/hea-bench (accessed on 8 July 2026), run directly in the browser at https://dfieser.github.io/hea-bench/ (accessed on 8 July 2026), and archived under the all-versions Zenodo concept DOI, which resolves to the latest release, at https://doi.org/10.5281/zenodo.20346287.

## Supplementary Materials

The following supporting information can be downloaded at: https://www.mdpi.com/article/10.3390/ma19143075/s1. Supplementary Methods: Miedema model constants, oxidation-state assignment by charge balance, and the coordination-number and spin-state conventions; Table S1: Element coverage of the alloy data tables; Table S2: Pair contributions to $\Delta H_{\mathrm{mix}}$ for Co_20_Cu_20_Fe_5_Mn_35_Ni_20_, in kJ mol^−1^; Table S3: Per-rule screening statistics on the 7127-composition public labeled union; Table S4: HEA-Bench descriptors as input features on the Pei 2020 labeled set (872 alloys, single-phase against multi-phase); Table S5: Mixing enthalpy (kJ mol^−1^) and Ω under two Miedema parametrization routes; Table S6: The 37-element atomic-property table used by the alloy descriptors, exactly as shipped; Table S7: Descriptors of Co_20_Cu_20_Fe_5_Mn_35_Ni_20_ computed by a language model (Claude Haiku 4.5) without and with the HEA-Bench tool, against the shipped values; Listing S1: Python library usage; Listing S2: Model Context Protocol server responses, including the in-band provenance fields and the worked Ω sensitivity exchange; Listing S3: Installation and Use Across the Four Surfaces.

## Abbreviations

The following abbreviations are used in this manuscript:

HEA	High-entropy alloy
HEO	High-entropy oxide
VEC	Valence-electron concentration
MCP	Model Context Protocol
DFT	Density functional theory
CALPHAD	Calculation of phase diagrams
