# Supplementary material for: HEA-Bench: An AI-Agent-Optimized Calculator of High-Entropy Alloy and Oxide Descriptors and Phase-Prediction Rules
Source: Materials (Basel). 2026 Jul 17;19(14):3075. doi: 10.3390/ma19143075 (PMC13413400; doi:10.3390/ma19143075)
Supplement: Supplementary file 1 [file materials-19-03075-s001.zip › materials-4429758-supplementary.pdf]

Supplementary Materials: Supplementary Materials: HEA-Bench: An AI-Agent-Optimized Calculator of High-Entropy Alloy and Oxide Descriptors and Phase-Prediction Rules

David Fieser [1,*], Unmanaa Dewanjee [1] and Anming Hu [1]

1. Supplementary Methods

This section records the model details and conventions behind the descriptors, at a depth that supports reproduction but would interrupt the main text. Every value below is fixed in the software and is regenerated only by the recorded provenance scripts.

1.1. Miedema Macroscopic-Atom Model

The chemical term of the formation enthalpy follows the macroscopic-atom model of de Boer and co-workers, in which each element carries an adjusted electronegativity φ^*, a boundary electron density n_{ws}, and a molar volume V. For an AB pair the interfacial amplitude Γ_{AB} defined in the main text combines an attractive electronegativity term in $(\Delta\varphi^*)^2$ and a repulsive electron-density term in $(\Delta n_{\mathrm{ws}}^{1/3})^2$, scaled by the empirical constants P and Q and corrected by a hybridization term R_{hyb} that applies only to pairs of a transition metal with a non-transition metal. The constants take the standard tabulated values, with P depending on the transition-metal character of the two partners and the ratio Q/P held fixed, so the only element-specific inputs are φ^*, n_{ws}, and V. The composition enters through surface concentrations $c_i^s = c_i V_i^{2/3} / \sum_k c_k V_k^{2/3}$, and the molar volumes themselves are corrected self-consistently for charge transfer between unlike neighbors. The solid-solution enthalpy adds an elastic size-mismatch term and a structural term keyed to the mean valence-electron count, and the amorphous enthalpy replaces those with a topological term, so the reported decomposition separates the chemical, elastic, structural, and topological contributions for each phase.

1.2. Oxidation-State Assignment by Charge Balance

For an oxide composition the program assigns cation oxidation states by exact charge balance before any radius is selected. Each cation is restricted to the oxidation states recorded for it in the vendored table, the search enumerates the allowed combinations weighted by the cation occupancies, and a combination is accepted only when the total cation charge equals twice the oxygen content of the formula unit. When more than one balanced assignment exists, the program prefers the assignment that uses each cation's most common state, and when none balances it raises a structured warning that names the cation set and the target charge rather than forcing an approximate fit. This ordering matters because a single wrong oxidation state changes the Shannon radius selected downstream and therefore every radius-based descriptor for that composition.

1.3. Coordination Numbers and Spin States

Shannon effective ionic radii are selected by oxidation state, by the coordination number of the crystallographic site in the chosen structure family, and by spin state where the table distinguishes one. The coordination numbers are six for the rock-salt cation site, twelve for the perovskite A site and six for its B site, eight for the fluorite cation site, and eight for the pyrochlore A site with six for its B site. The oxygen radius used in the Goldschmidt factor is the six-fold value of 1.40 Å. Where a cation has both a high-spin and a low-spin radius, the program uses the high-spin value by default and exposes the

choice as a parameter. The Ye φ index evaluates the hard-sphere excess entropy at the body-centered and face-centered packing fractions of 0.68 and 0.74 and averages the two, with the packing fraction also exposed as a parameter.

2. Element Coverage

Table S1 lists the elements covered by each alloy data table. Thirty-seven elements carry the full set of descriptors and rules. The Miedema pair-enthalpy table extends the mixing-enthalpy estimate to a further thirty-eight elements, for seventy-five in total. The oxide module covers ninety-four cations across the four structure families, shown in main-text Figure 2, with the full list in the repository.

Table S1. Element coverage of the alloy data tables. Thirty-seven elements carry full descriptor and rule support, and the Miedema pair-enthalpy table adds 38 more, for 75 in total. The oxide module covers 94 cations (main-text Figure 2).

Tier	Elements
Full support (37)	Li, Be, Mg, Al, Si, Ca, Sc, Ti, V, Cr, Mn, Fe, Co, Ni, Cu, Zn, Y, Zr, Nb, Mo, Ru, Rh, Pd, Ag, In, Sn, Hf, Ta, W, Re, Os, Ir, Pt, Au, La, Ce, Gd
Miedema only (+38)	H, B, C, N, Na, P, K, Ga, Ge, As, Rb, Sr, Tc, Cd, Sb, Cs, Ba, Hg, Tl, Pb, Bi, Po, At, Pr, Nd, Pm, Sm, Eu, Tb, Dy, Ho, Er, Tm, Yb, Lu, Th, Pa, U

3. Per-Pair Mixing-Enthalpy Decomposition

Table S2 gives the ten pair contributions $4\,\Delta H_{ij} c_i c_j$ to the mixing enthalpy of the near-ideal alloy $Co_{20}Cu_{20}Fe_5Mn_{35}Ni_{20}$ plotted in main-text Figure 8. The contributions sum to -0.52 kJ mol^{-1}, close enough to zero that the Yang–Zhang Ω sits near its singularity. The four manganese-containing pairs carry the largest magnitudes and have opposite signs, so a shift of a few kJ mol^{-1} in the manganese pair enthalpies, within the spread of published compilations, moves the sum substantially and swings Ω by an order of magnitude while leaving the single-phase verdict unchanged.

Table S2. Pair contributions to ΔH_{mix} for $Co_{20}Cu_{20}Fe_5Mn_{35}Ni_{20}$, in kJ mol^{-1}. Manganese pairs are marked.

Pair	Contains Mn	Contribution
Mn–Ni	yes	-2.24
Co–Mn	yes	-1.40
Fe–Ni	no	-0.08
Co–Fe	no	-0.04
Co–Ni	no	0.00
Fe–Mn	yes	0.00
Cu–Fe	no	$+0.52$
Cu–Ni	no	$+0.64$
Co–Cu	no	$+0.96$
Cu–Mn	yes	$+1.12$
**Total**		**-0.52**

4. Empirical-Rule Screening on Public Labeled Data

This section reports the success-and-failure statistics that quantify the weak-screen framing of the main text. The evaluation set is a union of three open datasets of experimentally observed high-entropy-alloy phases, Borg 2020, Pei 2020, and Peivaste 2023 [1–3], consolidated to 7,784 unique compositions on a normalized composition key. Removing

the 100 compositions on which the sources disagree and the 557 whose elements fall outside the descriptor table leaves 7,127 scored compositions. Borg 2020 and Pei 2020 are released under CC-BY-4.0 and Peivaste 2023 is redistributed pointer-only, so only aggregate statistics are reported here. Each rule that separates single-phase solid solutions from multi-phase microstructures is scored against the observed label, with the single-phase class as positive (Table S3). The canonical thresholds have high sensitivity and low specificity, so they flag most single-phase alloys but also pass many multi-phase ones, which is the measured basis for reporting the rules as screens with sources rather than as predictions. The valence-electron-concentration rule is a face-centered against body-centered structure typing among single-phase alloys, not a single-phase against multi-phase classifier, and reaches an accuracy of 0.675 on 3,567 typed alloys. The mixing-entropy rule is a descriptive high-entropy class boundary and is not a phase classifier.

Table S3. Per-rule screening statistics on the 7,127-composition public labeled union. Positive class is single-phase solid solution. Accuracy 95% confidence intervals are about ± 0.011. These are screen-quality statistics for the canonical published thresholds, not a performance claim for HEA-Bench, which computes the descriptors that the thresholds are applied to.

Rule	Criterion	n	Acc.	Sens.	Spec.	Youden J
Zhang δ [4]	$\delta < 6.5\,\%$	7127	0.581	0.989	0.149	$+0.138$
Yang Ω [5]	$\Omega > 1.1$	7127	0.549	0.950	0.125	$+0.074$
King Φ [6]	$\Phi > 1.0$	7127	0.489	0.827	0.130	-0.043
Ye φ [7]	$\varphi > 20$	7127	0.504	0.442	0.570	$+0.013$

5. Descriptors as Input Features for Standard Classifiers

To connect the descriptors to current phase-prediction practice, the five descriptors used by the recent classifier of Mandal and co-workers [8], the atomic-size mismatch δ, the mixing entropy ΔS_{mix}, the valence-electron concentration, the Miedema mixing enthalpy, and the electronegativity mismatch $\Delta\chi$, were computed with HEA-Bench for the Pei 2020 dataset [2]. After parsing, normalization, removal of duplicate compositions, and removal of compositions with elements outside the descriptor table, 872 alloys remain, 494 single-phase and 378 multi-phase. Two off-the-shelf scikit-learn classifiers were trained under five-fold stratified cross-validation with no tuning. This is an illustration that the descriptors carry the classification signal that modern models exploit, not a shipped capability of the tool and not a claim of state-of-the-art performance. Both classifiers score well above the majority-class baseline and above the same rules applied to the identical rows, and the random forest ranks the atomic-size mismatch the most important feature (Gini importances δ 0.37, ΔS_{mix} 0.36, mixing enthalpy 0.10, valence-electron concentration 0.10, $\Delta\chi$ 0.07), reproducing the δ-first ordering that Mandal and co-workers report from a leave-one-out analysis. Table S4 collects the accuracies.

Table S4. HEA-Bench descriptors as input features on the Pei 2020 labeled set (872 alloys, single-phase against multi-phase). The classifiers are stock scikit-learn defaults under five-fold stratified cross-validation. The rule rows are the same two thresholds scored on the identical rows for comparison.

Method	Accuracy
Majority-class baseline	0.567
Zhang $\delta < 6.5\,\%$ (same rows)	0.729
Yang $\Omega > 1.1$ (same rows)	0.560
Logistic regression, five descriptors	0.878 ± 0.032
Random forest, five descriptors	0.939 ± 0.011

6. Miedema Parametrization Spread

The main text (Section 4.5) shows the Ω sensitivity for one near-ideal alloy. Table S5 generalizes it across the eight Yang–Zhang panel alloys and the near-ideal alloy, comparing two Miedema routes that HEA-Bench can produce. Route A is the shipped matminer / Takeuchi–Inoue integer pair table used throughout the main text. Route B is the de Boer macroscopic-atom chemical solid-solution enthalpy computed from the browser and desktop parameters of the same tool. Both descend from de Boer 1988, so the comparison is a transparency exhibit for the tool's per-pair override rather than a claim that one parametrization predicts better than another. The mean melting temperature and the mixing entropy do not depend on the pair table, so the entire spread lives in the mixing enthalpy and propagates into Ω. The two routes agree to within about 1% in Ω for the transition-metal-only alloys and to 0.24 kJ mol^{-1} on the manganese pairs. They diverge most for the aluminium-bearing alloys, by up to about 5 kJ mol^{-1} and a factor of 2.2 in Ω, because the two compilations treat the aluminium hybridization term differently. Every rule verdict is unchanged across the two routes.

Table S5. Mixing enthalpy (kJ mol^{-1}) and Ω under two Miedema parametrization routes. A, shipped matminer / Takeuchi–Inoue integer pair table. B, de Boer macroscopic-atom recompute from the tool's browser parameters. The spread is dominated by the aluminium-bearing alloys.

	ΔH_{mix}		Ω	
**Alloy**	**A**	**B**	**A**	**B**
$CoCrFeNiMo_{0.3}$	−4.15	−4.14	6.00	6.02
$CoCrFeNiAl_{0.3}Mo_{0.1}$	−7.26	−9.81	3.39	2.51
$CoCrFeNiCuAlMo_{0.2}$	−4.47	−9.82	5.82	2.65
$Ti_{0.8}CoCrFeNiCu$	−6.75	−6.04	3.95	4.42
TiCoCrFeNiCu	−8.44	−7.62	3.17	3.51
$Ti_{1.5}CoCrFeNiAl$	−23.91	−27.86	1.08	0.93
CoCrFeNiCuAlMn	−5.63	−10.47	4.64	2.49
CrFeNiCuZr	−14.40	−13.99	1.71	1.76
$Co_{20}Cu_{20}Fe_5Mn_{35}Ni_{20}$	−0.52	−0.68	37.77	28.86

7. Element Property Table

Table S6 lists, element by element, the exact values in the 37-element property table that the alloy descriptors read, so a reader can reproduce any descriptor by hand and compare it against their own source data. The metallic radii follow the Goldschmidt twelve-coordinate convention, digitized from Housecroft and Sharpe [9] for the transition metals and from Teatum, Gschneidner, and Waber [10] for the rare earths. The melting points are the CRC Handbook reference values [11]. The electronegativities are on the Pauling scale [12], reproduced in the same CRC Handbook. The valence-electron counts use the transition-metal s-plus-d convention of Guo and Liu [13], so that, for example, copper is eleven and zinc is twelve. Boron and carbon are deliberately absent, because neither has a metallic radius on this convention and carbon has no melting point at one atmosphere, so including either would mix radius conventions inside the size-mismatch calculation.

8. A Grounded-versus-Unaided Agent Demonstration

To test whether grounding an agent in the tool changes what it produces, we posed one screening task to a language model twice, once unaided and once with the HEA-Bench tool, and compared the descriptor values each returned against the shipped values. The task was to compute all nine standard descriptors of the near-ideal alloy $Co_{20}Cu_{20}Fe_5Mn_{35}Ni_{20}$ (Table S7). Unaided, the model reproduced the descriptors that follow directly from tabulated element data, the mixing entropy, the valence-electron concentration, the mean

Table S6. The 37-element atomic-property table used by the alloy descriptors, exactly as shipped. r is the metallic atomic radius (pm), T_m the melting point (K), VEC the valence-electron count, and χ the Pauling electronegativity. Sources are given in the text above.

El.	r	T_m	VEC	χ	El.	r	T_m	VEC	χ
Ag	144.0	1234.93	11	1.93	Nb	147.0	2750.00	5	1.60
Al	143.0	933.47	3	1.61	Ni	125.0	1728.00	10	1.91
Au	144.0	1337.33	11	2.54	Os	135.0	3306.00	8	2.20
Be	112.0	1560.00	2	1.57	Pd	137.0	1828.00	10	2.20
Ca	197.0	1115.00	2	1.00	Pt	139.0	2041.40	10	2.28
Ce	182.0	1071.00	3	1.12	Re	137.0	3459.15	7	1.90
Co	125.0	1768.00	9	1.88	Rh	134.0	2237.00	9	2.28
Cr	129.0	2180.00	6	1.66	Ru	134.0	2607.00	8	2.20
Cu	128.0	1357.77	11	1.90	Sc	164.0	1814.00	3	1.36
Fe	126.0	1811.00	8	1.83	Si	111.0	1687.00	4	1.90
Gd	180.2	1585.00	3	1.20	Sn	158.0	505.08	4	1.96
Hf	159.0	2506.00	4	1.30	Ta	147.0	3290.00	5	1.50
In	167.0	429.70	3	1.78	Ti	147.0	1941.00	4	1.54
Ir	136.0	2719.00	9	2.20	V	135.0	2183.00	5	1.63
La	188.0	1193.00	3	1.10	W	141.0	3695.00	6	2.36
Li	157.0	453.65	1	0.98	Y	182.0	1799.00	3	1.22
Mg	160.0	923.15	2	1.31	Zn	137.0	692.68	12	1.65
Mn	135.7	1519.00	7	1.55	Zr	160.0	2128.00	4	1.33
Mo	139.0	2896.00	6	2.16					

melting temperature, and the electronegativity mismatch, but fabricated the five that require the Miedema pair table or a competing-phase model. Its mixing enthalpy was too negative by roughly a factor of twenty, its size mismatch too large by a factor of three, and its Ye φ too small by a factor of thirty, and it reported Ω as 1.6 with no indication that the value is numerically unstable here. The same model connected to the tool returned every one of these values exactly and surfaced the Ω instability. Extending this comparison into a systematic bare-against-grounded benchmark across many compositions, measuring the rate of fabricated values and verdict flips, is a natural next step.

Table S7. Descriptors of $Co_{20}Cu_{20}Fe_5Mn_{35}Ni_{20}$ computed by a language model (Claude Haiku 4.5) without and with the HEA-Bench tool, against the shipped values. Unaided, the model fabricated the five descriptors that need the Miedema pair table or a competing-phase model (marked †), several by more than an order of magnitude, and did not detect that Ω is numerically unstable here. With the tool it returned every value exactly.

Descriptor	Unaided	With HEA-Bench
ΔS_{mix} (J mol^{-1}K^{-1})	12.3	12.33
δ (%) †	12.3	3.67
VEC	8.85	8.85
mean T_m (K)	1593	1593
$\Delta\chi$	0.164	0.164
ΔH_{mix} (kJ mol^{-1}) †	−12	−0.52
Ω †	1.64	37.77
Φ (King) †	1.6	2.52
φ (Ye) †	0.88	28.31

9. Software Usage

9.1. Listing S1: Python Library Usage

The reference Python library installs from PyPI and has no dependencies. The descriptor functions take a composition mapping, formula strings are handled by the bundled

parser, and the rule modules return verdict strings, so the whole alloy workflow is a few lines. All example values below are pinned in the test suite.

```
import hea_bench as hb

cantor = hb.normalize(hb.parse_formula("CoCrFeMnNi"))
hb.smix(cantor)             # 13.381 J/(mol K) = R ln 5
hb.delta(cantor)            # 3.164 % atomic-size mismatch
hb.mixing_enthalpy(cantor)  # -4.16 kJ/mol (Miedema)

from hea_bench.rules import yang_omega
yang_omega.predict(cantor)  # 'single-phase'
```

The oxide module returns complete reports rather than single values, because the solved oxidation states and selected radii are part of the answer.

```
from hea_bench import oxides

pvk = oxides.describe_perovskite(
    {"Sr": 1}, {"Zr": 1, "Sn": 1, "Ti": 1, "Hf": 1, "Mn": 1})
pvk["descriptors"]["goldschmidt_t"]  # 0.979
pvk["verdicts"]["bartel"]            # 'perovskite'
```

9.2. Listing S2: Model Context Protocol Server Responses

The MCP server exposes the Python core as seven deterministic tools. Every returned value carries its unit, the citation key of its parametrization, and the software version, so the numbers an agent quotes in its reasoning trace can be audited rather than taken on trust. A fragment of a rule response for the Cantor alloy illustrates the shape.

```
{"rules": {"yang_omega": {
     "verdict": "single-phase",
     "value": 5.7937,
     "threshold": 1.1,
     "source": "Yang2012"}},
 "hea_bench_version": "2.0.4"}
```

The worked Ω sensitivity exchange of the main text proceeds as follows. One batch call evaluates the rules for the Cantor alloy and the near-ideal alloy together, and both pass the Yang–Zhang screen with an apparently comfortable margin.

```
{"input": "CoCrFeMnNi",
 "rules": {"yang_omega": {"verdict": "single-phase",
   "value": 5.7937, "threshold": 1.1, "source": "Yang2012"}}}
{"input": "Co20Cu20Fe5Mn35Ni20",
 "rules": {"yang_omega": {"verdict": "single-phase",
   "value": 37.7675, "threshold": 1.1, "source": "Yang2012"}}}
```

A second call, to the sensitivity tool, tests the margin and flags the divergence.

```
{"input": "Co20Cu20Fe5Mn35Ni20",
 "h_mix_kj_mol": -0.52, "omega": 37.7675,
 "dominant_element": "Mn",
 "h_mix_range_kj_mol": [-2.34, 1.30],
```

```
"omega_at_range_endpoints": [8.3928, 15.1070],
"diverges_within_range": true,
"advice": "The perturbation interval crosses h_mix = 0,
  so Omega is unbounded within the spread of published
  pair tables. Use the phase verdict, not the Omega
  magnitude."}
```


The same call on the Cantor alloy returns a bounded interval, Ω between 4.43 and 8.37 with no divergence flag, so the smaller of the two reported margins is in fact the trustworthy one.

9.3. Listing S3: Installation and Use Across the Four Surfaces

The same calculations are available from every surface. The Python library installs with `pip install hea-bench` and the optional agent server with `pip install "hea-bench[mcp]"`. The command line is invoked as `hea-bench`, the browser application runs at the hosted page or from a local copy of the repository with no build step, the desktop executable is a single offline file, and an MCP-capable client registers the `hea-bench-mcp` command to expose the seven tools to an agent.

References

1. Borg, C.K.H.; Frey, C.; Moh, J.; Pollock, T.M.; Gorsse, S.; Miracle, D.B.; Senkov, O.N.; Meredig, B.; Saal, J.E. Expanded dataset of mechanical properties and observed phases of multi-principal element alloys. *Scientific Data* **2020**, *7*, 430.
2. Pei, Z.; Yin, J.; Hawk, J.A.; Alman, D.E.; Gao, M.C. Machine-learning informed prediction of high-entropy solid solution formation: beyond the Hume-Rothery rules. *npj Computational Materials* **2020**, *6*, 50.
3. Peivaste, I.; Jossou, E.; Tiamiyu, A.A. Data-driven analysis and prediction of stable phases for high-entropy alloy design. *Scientific Reports* **2023**, *13*, 22556.
4. Zhang, Y.; Zhou, Y.J.; Lin, J.P.; Chen, G.L.; Liaw, P.K. Solid-solution phase formation rules for multi-component alloys. *Advanced engineering materials* **2008**, *10*, 534–538.
5. Yang, X.; Zhang, Y. Prediction of high-entropy stabilized solid-solution in multi-component alloys. *Materials Chemistry and Physics* **2012**, *132*, 233–238.
6. King, D.; Middleburgh, S.; McGregor, A.; Cortie, M. Predicting the formation and stability of single phase high-entropy alloys. *Acta Materialia* **2016**, *104*, 172–179.
7. Ye, Y.; Wang, Q.; Lu, J.t.; Liu, C.; Yang, Y. Design of high entropy alloys: A single-parameter thermodynamic rule. *Scripta Materialia* **2015**, *104*, 53–55.
8. Mandal, P.; Choudhury, A.; Mallick, A.B.; Ghosh, M. Phase prediction in high entropy alloys by various machine learning modules using thermodynamic and configurational parameters. *Metals and Materials International* **2023**, *29*, 38–52.
9. Housecroft, C.E.; Sharpe, A.G. *Inorganic Chemistry*, 4 ed.; Pearson: Harlow, UK, 2012.
10. Teatum, E.T.; Gschneidner, K.A.; Waber, J.T. Compilation of Calculated Data Useful in Predicting Metallurgical Behavior of the Elements in Binary Alloy Systems. Technical Report LA-4003, Los Alamos Scientific Laboratory, Los Alamos, NM, USA, 1968.
11. Haynes, W.M., Ed. *CRC Handbook of Chemistry and Physics*, 97 ed.; CRC Press: Boca Raton, FL, USA, 2016.
12. Pauling, L. *The Nature of the Chemical Bond and the Structure of Molecules and Crystals: An Introduction to Modern Structural Chemistry*, 3 ed.; Cornell University Press: Ithaca, NY, USA, 1960.
13. Sheng, G.; Liu, C.T. Phase stability in high entropy alloys: Formation of solid-solution phase or amorphous phase. *Progress in Natural Science: Materials International* **2011**, *21*, 433–446.
